# Association of Moderate‐to‐Severe Chronic Pain with Cognitive Decline and Alzheimer's Biomarkers in the Alzheimer's Disease Neuroimaging Initiative

**DOI:** 10.1002/alz70857_106768

**Published:** 2025-12-25

**Authors:** Tyler R. Bell, Carol E. Franz, Christine Fennema‐Notestine, Imanuel Lerman, Erin E. Sundermann, Shelby B Hughes, Jeremy A. Elman, William S. Kremen

**Affiliations:** ^1^ Center for Behavior Genetics of Aging, University of California, San Diego, La Jolla, CA, USA; ^2^ University of California San Diego, La Jolla, CA, USA; ^3^ University of San Diego California, San Diego, CA, USA; ^4^ University of California, San Diego, La Jolla, CA, USA

## Abstract

**Background:**

Chronic pain affects one in five older adults and is linked to increased risk for Alzheimer's disease (AD) dementia. Few studies have investigated associations between chronic pain and AD biomarkers, especially chronic pain at moderate‐to‐severe levels considered clinically significant.

**Method:**

We included 2487 participants without baseline dementia from the Alzheimer's Disease Neuroimaging Initiative (ADNI). At baseline, participants were categorized by absence/presence of moderate‐to‐severe chronic pain, defined as persistent/recurring pain with onset >3 months prior. For sensitivity analyses, we categorized people based on the absence/presence of chronic pain at mild levels. Cognitive outcomes included the Clinical Dementia Rating Scale Sum of Boxes (CDR‐SB), episodic memory, and executive function (minimal *n* = 1328, mean follow‐up‐years=1.49, SD=2.78). AD biomarkers included cerebrospinal fluid levels of Aβ42, total tau, and phosphorylated tau (minimal *n* = 1057, mean follow‐up‐years = .53, SD=1.24); and structural MRI (AD structural brain signature, hippocampal volume, entorhinal cortical thickness, and whole brain volume, minimal *n* = 2136, mean follow‐up‐years=1.11, SD=2.28). Mixed‐effects models assessed differences in baseline levels and change in each outcome between pain groups, and mediation analyses explored Aβ and neurodegeneration pathways. Covariates included baseline age, sex, APOE ε4 status, depressive symptoms, and opioid use (intracranial volume for structural MRI outcomes).

**Result:**

Moderate‐to‐severe chronic pain relative to mild or none was associated with accelerated decline in global cognition (increase in CDR‐SB: b = .05), memory, executive function (b=‐.04), and whole brain volume (b=‐.02), and faster accumulation of Aβ pathology (CSF Aβ42 decline: b=‐.07, all *p*'s < .05). It was not associated with neurodegeneration in AD brain signature regions, hippocampal volume, or entorhinal cortex (all *p*s > .05). Accumulation of Aβ pathology (absolute b's range = .03 to .05) and whole brain volume loss (absolute b's range = .001‐.002) mediated associations of moderate‐to‐severe chronic pain with cognitive decline, with Aβ also mediating moderate‐to‐severe chronic pain's association with brain volume loss (b=‐.01, all *p*s < .05). Mild chronic pain relative to none did not significantly relate to cognitive or biomarker outcomes (all *p*s > .05).

**Conclusion:**

Moderate‐to‐severe chronic pain contributes to accelerated cognitive decline and general neurodegeneration, primarily via Aβ. These findings highlight the need to consider moderate‐to‐severe chronic pain, rather than just presence/absence of chronic pain in AD risk assessments and interventions.

